# Comparative Evaluation of TiO_2_ Nanoparticle Addition and Postcuring Time on the Flexural Properties and Hardness of Additively Fabricated Denture Base Resins

**DOI:** 10.3390/nano13233061

**Published:** 2023-11-30

**Authors:** Maram A. AlGhamdi, Shaimaa M. Fouda, Noha Taymour, Sultan Akhtar, Soban Q. Khan, Mohamed S. Ali, Ahmed M. Elakel, Essam A. Nassar, Mohammed M. Gad

**Affiliations:** 1 Department of Substitutive Dental Sciences, College of Dentistry, Imam Abdulrahman Bin Faisal University, P.O. Box 1982, Dammam 31441, Saudi Arabia; smfouda@iau.edu.sa (S.M.F.); ntyoussef@iau.edu.sa (N.T.); msali@iau.edu.sa (M.S.A.); 2 Department of Biophysics, Institute for Research and Medical Consultations (IRMC), Imam Abdulrahman Bin Faisal University, P.O. Box 1982, Dammam 31441, Saudi Arabia; suakhtar@iau.edu.sa; 3 Department of Dental Education, College of Dentistry, Imam Abdulrahman Bin Faisal University, P.O. Box 1982, Dammam 31441, Saudi Arabia; sqkhan@iau.edu.sa; 4 Department of Preventive Dental Sciences, College of Dentistry, Imam Abdulrahman Bin Faisal University, P.O. Box 1982, Dammam 31441, Saudi Arabia; amelakel@iau.edu.sa (A.M.E.); enassar@iau.edu.sa (E.A.N.)

**Keywords:** 3D printing, nanoparticles, mechanical testing, hardness, post-curing time

## Abstract

Three-dimensionally (3D)-printed fabricated denture bases have shown inferior strength to conventional and subtractively fabricated ones. Several factors could significantly improve the strength of 3D-printed denture base resin, including the addition of nanoparticles and post-curing factors. This study evaluated the effect of TiO_2_ nanoparticle (TNP) addition and the post-curing time (PCT) on the flexural properties and hardness of three-dimensionally (3D)-printed denture base resins. A total of 360 specimens were fabricated, with 180 specimens from each type of resin. For evaluating the flexural properties, bar-shaped specimens measuring 64 × 10 × 3.3 mm were used, while, for the hardness testing, disc-shaped specimens measuring 15 × 2 mm were employed. The two 3D-printed resins utilized in this study were Asiga (DentaBASE) and NextDent (Vertex Dental B.V). Each resin was modified by adding TNPs at 1% and 2% concentrations, forming two groups and an additional unmodified group. Each group was divided into three subgroups according to the PCT (15, 60, and 90 min). All the specimens were subjected to artificial aging (5000 cycles), followed by testing of the flexural strength and elastic modulus using a universal testing machine, and the hardness using the Vickers hardness test. A three-way ANOVA was used for the data analysis, and a post hoc Tukey’s test was used for the pairwise comparisons (α = 0.05). Scanning electron microscopy (SEM) was used for the fracture surface analysis. The addition of the TNPs increased the flexural strength in comparison to the unmodified groups (*p* < 0.001), while there was no significant difference in the elastic modulus and hardness with the 1% TNP concentration. Among the TNP groups, the 2% TNP concentration significantly decreased the elastic modulus and hardness (*p* < 0.001). The SEM showed a homogenous distribution of the TNPs, and the more irregular fracture surface displayed ductile fractures. The PCT significantly increased the flexural strength, elastic modulus, and hardness (*p* < 0.001), and this increase was time-dependent. The three-way ANOVA results revealed a significant difference between the material types, TNP concentrations, and PCT interactions (*p* < 0.001). Both concentrations of the TNPs increased the flexural strength, while the 2% TNP concentration decreased the elastic modulus and hardness of the 3D-printed nanocomposites. The flexural strength and hardness increased as the PCT increased. The material type, TNP concentration, and PCT are important factors that affect the strength of 3D-printed nanocomposites and could improve their mechanical performance.

## 1. Introduction

Edentulous patients are commonly treated with complete dentures to restore esthetics and function. A complete denture includes the denture teeth and denture base, which is mostly fabricated from poly methyl methacrylate (PMMA) [1]. PMMA is a biocompatible, esthetic, lightweight, and easily fabricated and repaired material. Nevertheless, it has low mechanical strength, low wear resistance, and is subject to polymerization shrinkage, in addition to microbial adhesion and discoloration with exposure to different food and drinks [2,3]. The new technologies of computer-aided designing and computer aided manufacturing (CAD-CAM) have been increasingly used for denture construction over the past 10 years to avoid the drawbacks of conventional methods and materials [4,5]. The CAD-CAM fabrication of denture bases could be achieved using a subtractive method, in which they are milled from a prefabricated disc, or an additive method, in which they are built in layers using three-dimensional (3D) printing and photopolymerized resins [6]. Studies have shown that milled resin has a higher strength than 3D-printed resins [5], but subtractive technology is more expensive and produces more material waste than 3D printing [7].

The fabrication of denture base resin using 3D printing technology has recently increased. It has numerous advantages [8], including precision and accuracy, which can lead to improved denture fit and function. In addition, the faster production time and reduced material waste decrease the cost associated with the fabrication process [9,10]. Two common 3D printing technologies are used for denture fabrication: stereolithography (SLA) and digital light processing (DLP) [11,12]. SLA uses a laser to cure liquid resin incrementally, while DLP utilizes a digital light projector to cure the resin [6]. DLP is faster than SLA, as it can cure an entire layer at once, while SLA must cure each layer point by point with a laser. Accordingly, SLA offers a higher resolution and more precise details than DLP due to the use of a laser. However, DLP printers are less expensive than SLA printers, rendering them a better choice for small dental offices [13].

Flexural strength is defined as the material’s capacity to withstand bending or fracture when subjected to an applied force. Three-dimensionally printed denture base resins have a lower flexural strength than conventionally fabricated and milled resins, which can affect the durability and lifespan of the denture [1,14]. The surface microhardness is another important characteristic of denture base materials because it refers to the material’s ability to resist indentation or scratching under a defined load. Thus, a higher surface hardness indicates a more durable and wear-resistant material. Several factors can influence the surface microhardness of 3D-printed resin, including the resin type, printing parameters, and postprocessing techniques [15,16]. 

Normally, the denture base is subjected to various stresses in the oral cavity; therefore, testing the specimens in simulated oral conditions would increase the reliability of the results after aging and decrease the study limitations [16]. During thermal cycling, the recurrent expansion and shrinkage of the polymeric network negatively affect the strength. In addition, the water sorption process and the rate of absorption increase with temperature, leading to polymer hydrolysis [17]. Because of the polarity inherent in resin materials, water has the ability to permeate the resin’s internal structure, causing monomers and other additives to migrate outward. Furthermore, water infiltrates between the polymer chains gaps, pushing these chains apart and weakening the intermolecular forces, transitioning them into weaker “van der Waals bonds”. This, in turn, initiates cracks within the material, causes it to expand, and ultimately reduces its mechanical properties [18]. Over time, certain components leak out, and the ingress of water causes the swelling, hydrolysis, and degradation of the cross-linked polymer [19]. All of these factors collectively deteriorate the resin’s mechanical properties

It is crucial to emphasize that the properties of 3D-printed denture base resin may differ depending on the specific material used, the 3D printing technology employed, and the postprocessing process. Some studies have found that certain 3D-printed denture base resins can exhibit comparable or even superior properties to conventionally fabricated and milled resins, especially when optimized printing parameters and postprocessing techniques are used [16,20,21,22]. The properties of 3D-printable resin, including strength, flexibility, and biocompatibility, are also affected by the chemical composition of the resin [23]. Moreover, the parameters for printing can influence the quality and accuracy of the resultant resin [24]. Postprocessing procedures, such as polishing, coating, or curing, can further modify the characteristics of the 3D-printed resin. Proper postprocessing could improve the surface finishing, strength, and biocompatibility of the resultant resin. Three-dimensional printing technology (SLA or DLP) can also affect the resolution, speed, and accuracy of the printed resin [9]. The careful consideration and optimization of these factors are necessary to ensure that 3D-printed resin has the desired characteristics for its expected purpose or application [25].

The impregnation/inclusion of nanoparticles into 3D-printable resin can potentially improve the characteristics of the printable resin that are dependent on the specific type and concentration of the nanoparticles [26,27]. Incorporating titanium dioxide nanoparticles (TNPs) into resin can enhance its strength, stiffness, wear resistance, and biocompatibility. The application of TNPs in dentistry has increased due to its high corrosion resistance, strength, and refractive index [28,29,30]. In addition, the cytotoxicity and biocompatibility of TNPs have been tested in multiple studies, which have shown its excellent biocompatibility and chemical stability [29,31,32]. A recent study reported that the addition of TNPs to 3D-printed denture base resin produced a biocompatible nanocomposite that has an antifungal effect against *Candida albicans.* Raj et al. [32] tested the toxicity of TNP-modified heat-cured acrylic resin after 1 and 7 days, and reported a low toxicity of the nanocomposite that decreased with time, confirming the biocompatibility of TNPs. Other studies found similar results of a decreased toxicity with aging, that might result from the release of free molecules into the aqueous media [33,34]. 

The nanoparticles can act as reinforcing agents, providing additional strength and stiffness to the printed material. Furthermore, nanoparticles can reduce the surface roughness and increase wear resistance of the printed material [6]. It was reported that nanofiller addition of a certain amount is effective and increases the mechanical performance, while concentrations higher than the saturation amount weaken the composite properties [21,35]. Three-dimensionally printed resin contains filler, to a limit, maintaining normal viscosity and a high degree of conversion; therefore, the concentration of any additive must be considered. TNPs have been added to 3D-printed resins for their antimicrobial activities. Totu et al. [36] added TNPs at different concentrations (0.2, 0.4, 0.6, 1, 2.5 by weight %) to serve as antimicrobial agents, and found that this nanocomposite is suitable for printing and has antimicrobial activity; however, biocompatibility and mechanical testing to prove this nanocomposite prior to clinical usage was recommended. Liu et al. investigated the effects of TNPs at different concentrations on the mechanical properties of 3D-printable materials, and found the best results with 1.5% TNPs [35]. The addition of 1% TNPs was also reported in a previous study as the optimum ratio to improve both the mechanical and antibacterial properties of 3D-printed PMMA composite resins [31]. Moreover, Tandra et al. and Karci et al. found that 1% TNPs increased the flexural strength of denture base resin [37,38]. However, Harini et al. showed that 1%, 2%, and 5% TNP concentrations increased the flexural strength of PMMA. The improvement in flexural strength might have resulted from the small filler size, that enhances binding to the polymer matrix, thus improving the resin strength [39]. On the contrary, some studies have found that the addition of TNPs at various concentrations that ranged from 1–5% reduced the flexural strength of denture base resin [40,41]. 

Post-curing is a common procedure in 3D printing to enhance the physio-mechanical properties of the additively printed resin [42]. Post-curing involves exposing the printed resin to additional UV light or heat after printing to fully cure the material and improve its final properties [43]. The post-curing time (PCT) can improve the printed resin strength by fully curing the material and reducing the risk of cracking or fracturing. PCT can also enhance the biocompatibility of the printed resin by decreasing the amount of residual monomers or other potentially harmful chemicals that may be present in the uncured resin [44].

There has been a lack of studies examining the influence of TNPs and PCT to enhance the properties of 3D-printed resin beyond what can be achieved by each technique alone. The aim of this study was to evaluate the use of TNPs and PCT as techniques for enhancing the properties of 3D-printable resin by investigating the effect of TNP incorporation with different PCTs on the strength of 3D-printed resin.

The first study’s null hypothesis is that the incorporation of TNPs into 3D-printed resin would not improve the strength of the resin. The second hypothesis is that different PCTs with incorporated TNPs will not increase the flexural strength, elastic modulus, or hardness of the printable resin.

## 2. Materials and Methods

The sample size of the present study was determined according to the results obtained from a prior study [45]. The recorded flexural strength mean values (SD) of denture base resins with different concentrations of TNPs (0%, 1%, and 2.5%) were 88.6 (6.2), 55.03 (3.1), and 42.4 (3.7), respectively. For the calculation of sample size, power analysis was performed. The parameters involved for the calculation were the power (80%) and confidence interval (95%), and the level of significance was set as 0.05. Therefore, based on the calculations, 10 samples per group were needed.

According to ISO 20795-1:2013 [46], flexural strength specimens were prepared with dimensions of 64 × 10 × 3.3 mm, while a disc shape of 15 × 2 mm was fabricated for hardness measurement, using the AutoCAD software (123D design, Autodesk, version 2.2.14, San Francisko, CA, USA). Subsequently, these designs were converted into standard tessellation language (STL) files. The STL file was imported to two printers with two different printing technologies: DLP for ASIGA, (ASIGA DentaBASE, Erfurt, Germany) and SLA for NextDent (Next Dent Base; Denture 3D+, 3D, systems, Vertex Dental B.V., Soesterberg, The Netherlands).

**Figure 1:**
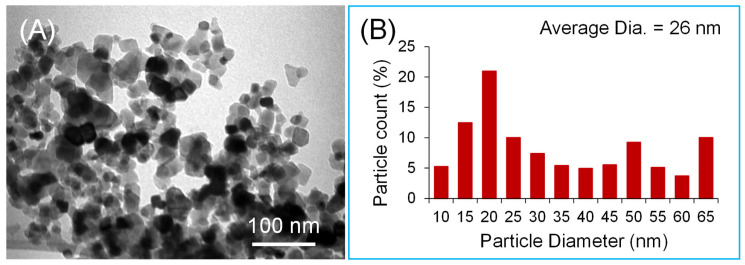
TEM image (**A**) and corresponding size histogram (**B**) showing the size distribution and the average size of the TNPs.

The size, shape, and structure of the TNPs were investigated using transmission electron microscopy (TEM) (Morgagni 268, FEI; operated at 80 kV) (Figure 1). For TEM, the sample suspension was deposited onto a TEM copper grid with carbon films. The electronic images were recorded at different magnifications to determine the morphology and structure of the nanoparticles. The average particle size was approximately 26 nm.

TNPs (Sigma–Aldrich, Co., St Louis, MO, USA) were silanated following the same process detailed in an earlier study [47]. The silanated TNPs were added to fluid resins at two concentrations, 1% and 2% by wt. Two 3D-printable resins were selected and studied in the present research: ASIGA and NextDent [Table 1]. A digital balance was used to determine the resin/TNP proportions. Before adding NP, each resin container was shaken for a homogenous distribution of resin compositions according to the manufacturer’s recommendations. In separate containers, 2 per resin type and 1 per concentration were prepared containing resin with 1% and 2% TNPs. According to the TNP concentration, each resin was divided into 3 groups: unmodified, 1% TNPs, and 2% TNPs. To avoid light contamination, the prepared nanocomposite mixtures were kept in a dark box away from direct light at room temperature.

Before printing, each container was shaken for 1 h using the LC 3D Mixer (NextDent, Soesterberg, The Netherlands) and then poured into the resin tank. The STL file with support design was imported to each printer followed by printing order according to the following parameters (90-degree printing orientations, 50 µm layer thickness, and 405 nm wavelength UV light). After printing, the specimens were cleaned of any remaining uncured resin using isopropyl alcohol and then subjected to post-curing for additional polymerization. According to PCT, each concentration group was subdivided further into 3 subgroups of 15, 60, and 90 min (*n* = 10). Post-curing was performed in the post-curing devices ASIGA Flash (ASIGA, Sydney, Australia) and LC-3DPrint Box, NextDent, Soesterberg, The Netherlands), according to the manufacturer’s recommendations. Afterwards, the support resins were removed, and the specimens were polished with an automated polishing machine (Metaserv 250 grinder-polisher; Buehler GmbH, Lake Bluff, IL, USA) using 1200-grit sandpaper (MicroCut PSA; Buehler, IL, USA) for 5 min at 100 rpm in wet conditions. The specimens were then kept for 2 days in distilled water at 37 °C, followed by thermal cycling (Thermocycler, THE-1100/THE-1200, SD Mechatronik GMBH Miesbacher Str. 34 83,620 Feldkirchen- Westerham, Germany) for 5000 cycles, with soaking for 5 s between 5 and 55 °C/30 s of dwell time.

A three-point bending test was used to evaluate the flexural strength and elastic modulus. The specimens were placed on two supports separated by 50 mm, on a universal testing machine (Instron Model 8871; Instron Corp., Canton, MA, USA), the crosshead speed was 5 mm/min, and a 5 kN load was applied at the center of the specimen until fracture [48]. The following equations were used to measure the values of flexural strength and elastic modulus (MPa):

FS = 3 WL/2bh^2^ and EM = FL^3^/4bh^3^d (1)

FE = flexural strength, W = fracture load (N), L = 50 mm i (distance between the supports), b = width of the specimen, h= thickness of the specimen. E = elastic modulus (MPa), F = is the load (N) at a convenient point (p) in the straight line of the tension/deformation curve (elastic deformation), and (d) is the deflection at that point (p).

A scanning electron microscope (SEM) (TESCAN VEGA 3, Czech Republic; operated at 20 kV) was used to analyze the fractured surfaces. The samples were gold-coated using a gold coating machine and then analyzed for fracture type and NP distributions under different magnifications.

Fourier transform infrared (FTIR) analysis was performed to evaluate the bond between TNPs and the matrix using transmission spectroscopy (Hartmann & Braun, MB-series). The FTIR spectra were recorded between the 4000 and 400 cm^−1^ wavenumber region at a resolution of 4 cm^−1^, as explained in a previous study [32]. The bonding and bands for the 3D-printed resin (in %) was determined by evaluating the ratio of the double carbon bond peaks at two frequencies: the stretch of the aliphatic frequency at 1637 cm^−1^ against the reference aromatic frequency at 1608 cm^−1^.

A Vickers microhardness test was conducted utilizing a Vickers tester (Tukon 1102, Wilson Hardness, ITW Test & Measurement, Shanghai, China) with a 25-gf load applied for 30 s at the specimen’s center with a diamond pyramid indenter. Subsequently, the diagonals of the resulting indentations were measured under a microscope to determine the hardness. Each specimen underwent a total of 3 indentations made at distinct locations and the average values for each specimen were calculated [48].

### Statistical Analysis

The Statistical Package of Social Sciences (SPSS v.23) was used for data analysis. Means and standard deviations were computed for the descriptive analysis of the data. Insignificant results from the Shapiro–Wilk test proved that the data were normally distributed. Therefore, parametric tests were used for inferential analysis. One-way ANOVA was used to study the effect of time or concentration levels on the tested properties of the resins. In addition, Tukey’s post hoc test was used for pairwise comparisons. Three-way ANOVA was used to assess the interaction effect of time, concentration, and resins. All *p* values less than 0.05 were considered statistically significant.

## 3. Results

**Figure 2:**
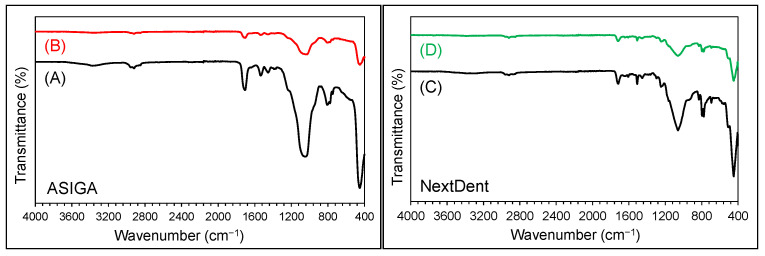
FTIR spectra of two ASIAGA and two ND specimens. (**A**) pure ASIGA (60 min), (**B**) ASI-GA/0.25%TiO_2_ (60 min), (**C**) pure NextDent (60 min), and (**D**) ND/0.25%TiO_2_ (60 min). The im-portant bands are appeared in each spectrum.

The FTIR spectra for the specimens from all the groups of the pure and modified 3D-printed resin with TNP showed similar types of bands and characteristic features. This proves that the addition of NPs did not change the chain structure of the nanocomposite, and only varied the intensity of the bands. The characteristic bands for each specimen are labelled and identified in Figure 2.

**Figure 3:**
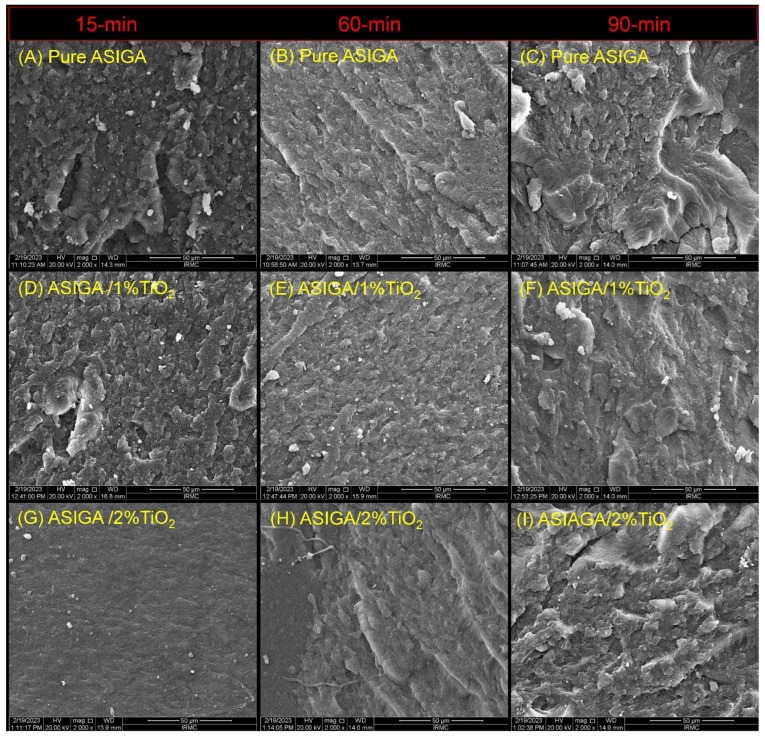
Representative SEM images of the fractured surfaces after mechanical testing for ASIGA specimens. Images were ordered horizontally according to PCT while vertically according TNPs concentrations. (**A**) pure ASIGA (15 min), (**B**) pure ASIGA (60 min), (**C**) pure ASIGA (90 min); (**D**) ASIGA/1%TiO_2_ (15 min), (**E**) ASIGA/1%TiO_2_ (60 min), (**F**) ASIGA/1%TiO_2_ (90 min); (**G**) ASIGA/2%TiO_2_ (15 min), (**H**) ASIGA/2%TiO_2_ (60 min), (**I**) ASIGA/2%TiO_2_ (90 min). All scale bars are 50 µm.

**Figure 4:**
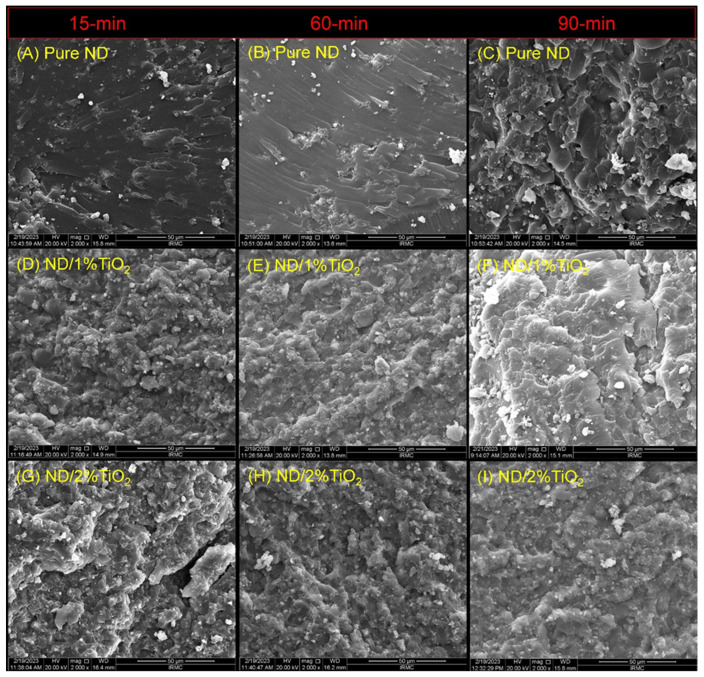
Representative SEM images of the fractured surfaces after mechanical testing for NextDent specimens. Images were ordered horizontally according to PCT while vertically according TNPs concentrations. (**A**) pure ND (15 min), (**B**) pure ND (60 min), (**C**) pure ND (90 min); (**D**) ND/1%TiO_2_ (15 min), (**E**) ND/1%TiO_2_ (60 min), (**F**) ND/1%TiO_2_ (90 min); (**G**) ND/2%TiO_2_ (15 min), (**H**) ND/2%TiO_2_ (60 min), (**I**) ND/2%TiO_2_ (90 min). All scale bars are 50 µm.

The SEM findings are displayed in Figure 3 and Figure 4. For the ASIGA specimen [Figure 3], the effect of the TNPs is the same as the pure sample when comparing the concentrations regarding the time, respectively, which shows a variation between the presence of small voids and some faint lamellae, except for the 2% sample at 15 min, with the absence of voids and lamellae (exhibited brittle to immediate fracture mode). Regarding the time effect, as time increased, irregularities with sharp lamellae were observed and were more obvious at 90 min regardless of the TNP concentration (exhibited a ductile fracture mode). For NextDent [Figure 4], all groups showed similar features, in which cracks and lamellae were distributed and concentrated evenly in the groups with and without NPs. The variations were in the surface irregularity, which was mainly related to the post-curing time; as the time increased, the irregularity increased, especially for the 1%NT sample at 90 min. All the fracture modes ranged from an intermediate (at 15 min) to ductile mode of fracture at 60 and 90 min. Additionally, the appearance of TNP clusters are considered a feature of the TNP-modified groups.

**Figure 5:**
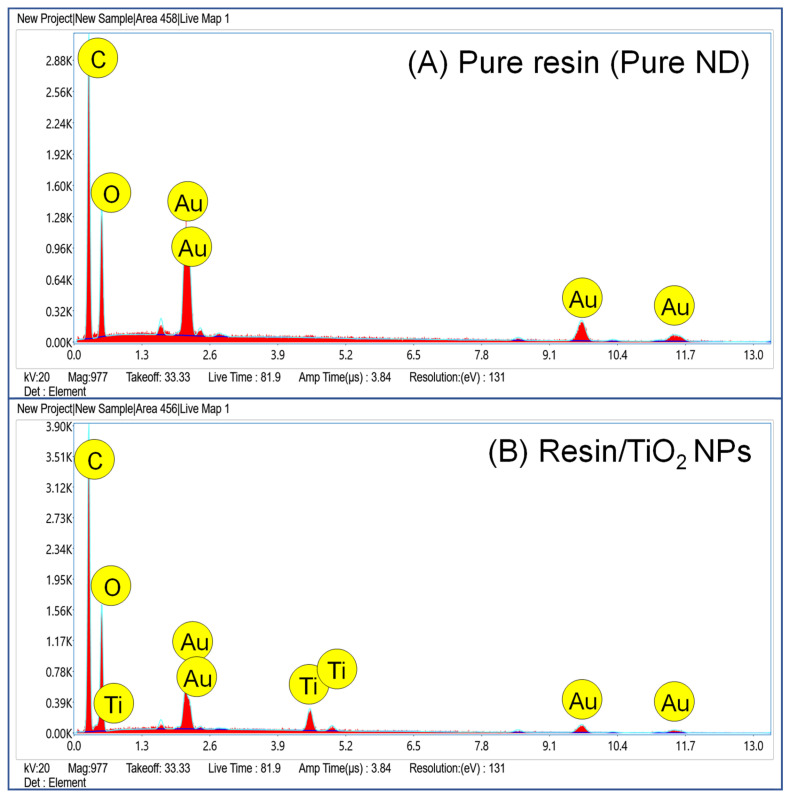
EDX spectra of (**A**) pure and (**B**) TiO_2_-enhanced resin (ND/2%TiO_2_). The spectrum of ND/2%TiO_2_ shows the peaks for incorporated Ti. Au peaks appeared due to gold coating.

In the SEM figures, the particles seen on the surface of the resin are identified as TNP aggregates or TNP agglomerates, and appeared during the fracturing of the specimen. The number of these particulates is higher as the concentration of TNPs was increased in the resin. The EDX (energy dispersive X-rays spectroscopy) of the fractured surface verified the presence of TNPs, as confirmed by the appearance of a Ti peak at ~4.5 Kev. The spectrum of the TiO_2_ resin is shown below (Figure 5). 

The mean values for the flexural strength, modulus of elasticity, and hardness were determined for each time point and for each concentration level for the ASIGA resin [Table 2]. The analysis of the variation in the average flexural strength due to the variation in PCT revealed that the average flexural strength increased with increased PCT and was highest at 90 min at any concentration level. The variation was statistically significant, with a *p* value less than 0.001. Similarly, the flexural strength was found to be highest at the 2% concentration level at any point in time. However, a marked difference was detected only when the time was 60 min (*p* < 0.001). The pairwise comparison showed that only one pair (0% vs. 1%) did not show a significant difference in their mean.

Regarding the elastic modulus, the highest elastic modulus was found at 60 min when the concentration was 0% and 2%, and at 90 min when the concentration was 1%. However, significant variation in the averages was found only when the concentration was 1% (*p* = 0.035). The pairwise comparison provided only one pair (60 min vs. 90 min) with significantly different means (*p* < 0.05). Similarly, the evaluation of the effect of the concentration levels at each point in time showed that, at 15 and 90 min, the 1% concentration provided the highest average elastic modulus, while, at 60 min, the elastic modulus was found to be highest at the 0% concentration. However, significance in the variation due to the concentration levels was found only at 90 min (*p* = 0.022). The pairwise comparison showed that the pair (1% vs. 2%) had a statistically significant difference in their means (*p* < 0.05).

Regarding the hardness, the highest mean hardness was found at 90 min at any given level of concentration. However, significant variation in the averages was found only when the concentration was 0% (*p* = 0.038). The pairwise comparison provided only one pair (15 min vs. 60 min) with significantly different means (*p* < 0.05). Similarly, evaluating the effect of the concentration levels at each point in time showed that, at 15 min, the 1% concentration provided the highest average hardness, while, at 60 and 90 min, the hardness was highest at the 0% concentration. However, significance in the variation due to the concentration levels was found only at 90 min (*p* = 0.043). The pairwise comparison showed that the pair (0% vs. 1%) had a statistically significant difference in their means (*p* < 0.05).

Table 2 presents the mean values of the flexural strength, elastic modulus, and hardness at each point in time and at each concentration level for the NextDent resin. The analysis of the variation in the average flexural strength due to the variation in time revealed that the average flexural strength was highest at 90 min at any concentration level. The variation was statistically significant, with a *p* value less than 0.001. The pairwise comparison revealed that at 2%, one pair (15 min vs. 60 min) did not have a significant difference in their means [Table 2]. Similarly, the flexural strength was found to be highest at the 2% concentration level at any point in time. A significant difference was observed at each time point (*p* < 0.001). The pairwise comparison revealed that, at 60 and 90 min, only one pair (1% vs. 2%) did not have a significant difference in their means.

Regarding the elastic modulus, when the time was 15 min, the highest elastic modulus was observed at the 0% concentration level. When the time was 60 and 90 min, the highest average was found at the 1% concentration level. However, a significant variation in the averages was found only when the concentration was 1% and 2% (*p* = 0.000 and *p* = 0.000, respectively). The pairwise comparison provided only one pair (60 min vs. 90 min) at each concentration level having significantly different means (*p* < 0.05). Similarly, the evaluation of the effect of the concentration levels at each point in time showed that a significance in the variation due to the concentration levels was found only at 15 and 60 min (*p* = 0.012 and *p* = 0.02, respectively). The pairwise comparison showed that, at 15 min, the pair (0% vs. 1%) had a statistically significant difference in their means (*p* < 0.05). At 60 min, only 0% vs. 1% had a statistically significant difference in their means (*p* < 0.05) [Table 2].

In the case of the hardness, the highest average hardness was found at 90 min when the concentration was 0% and 1%. For the 1% concentration, the highest average hardness was found at 15 min. However, a significant variation in the averages was found only when the concentration was 0% (*p* = 0.029). The pairwise comparison provided only one pair (15 min vs. 90 min) with significantly different means (*p* < 0.05). Similarly, the evaluation of the effect of the concentration levels at each point in time showed that, at 15 min, the 2% concentration provided the highest average hardness, while, at 60 and 90 min, the hardness was highest at the 1% concentration level. However, the significance in the variation due to the concentration levels was not found at any point in time.

The interacting effects of two and three factors were analyzed by using a three-way ANOVA for each tested property [Table 3]. It was found that, in terms of the flexural strength, only the combined effect of the concentration level and type of resin (*p* = 0.005) had a significant effect. In addition, in the case of the elastic modulus, the mutual effect of time with the type of resin (*p* = 0.000), the concentration with the type of resin (*p* = 0.015) and time with the concentration with the type of resin (*p* = 0.008) had a significant effect on the elastic modulus. For the hardness, the combined effect of the concentration and the type of resin was found to be statistically significant (*p* = 0.047).

Table 4 shows the material comparison in terms of the concentration and fixed post-curing time. For FS, only the pure ASIGA resin showed higher values compared with the NextDent resin, while the NT groups showed higher values for the NextDent over ASIGA resins, and the 2%NextDent resin showed a higher FS in terms of their respective times. All the pairwise comparisons were nonsignificant except for the elastic modulus, the same behaviors were observed for NextDent values higher than the ASIGA values in terms of their respective times, and all were nonsignificant except for the 1%NTASIGA vs. 1%NT-NextDent resin at 60 and 90 min (*p* < 0.001) and the 2%NTASIGA vs. 2%NT-NextDent resin at 90 min (*p* < 0.001). For the hardness, the NextDent resin also showed higher values compared to the ASIGA resin in terms of their respective times and concentrations; however, all the pairwise comparisons were nonsignificant except for the 2%NTASIGA vs. 2%NT-NextDent resin at 60 and 90 min (*p* < 0.001).

## 4. Discussion

This study tested the strength of nanocomposites in addition to the effect of the PCT. According to the results, the study’s first hypothesis was partially accepted, as the addition of the TNPs showed variable effetcs on the FS, elastic modulus, and hardness of the printed resin depending on the nanoparticle concentration. The PCT increased the flexural strength and hardness; therefore, the second hypothesis was rejected. 

The specimens in this study were subjected to thermal stress with 5000 cycles, equivalent to 6 months of clinical use [19]. All the specimens were subjected to thermal stress prior to testing to simulate the effects of aging.

Many studies [49,50,51] tested the effect of the PCT on the flexural strength and found a correlation between the post-curing time and flexural strength. The printed resin has a green appearance when it is not completely polymerized, and thus contains a high amount of residual monomer [49]. For complete polymerization to occur, each manufacturer recommended the minimum appropriate PCT; however, the maximum PCT was not reported [51]. Different PCTs have been suggested in the literature, ranging from 15 to 120 min. Studies have shown a positive correlation between the strength of 3D-printable resin and PCT [6,51,52]. This is in accordance with the present results of an increased flexural strength with an increased PCT, as the flexural strength increased with a 60-min and 90-min PCT. This is in agreement with previous studies that evaluated the effect of an increased PCT up to 120 min and found that the flexural strength exhibited a gradual increase up to a post-curing time of 120 min, and beyond 60 min of post-curing time, there were no notable significant differences [29,49]. Aati et al. [50] used short PCTs (0, 5, 10, and 20 min), which are close to the recommended time; however, the study reported an improvement in the flexural strength as the post-curing time increased. Additionally, Li et al. reported a higher flexural strength with increased PCT, and concluded that, for clinical application, an appropriate post-curing method would optimize the flexural strength of the 3D-printed denture [29].

Photosensitive resins suitable for 3D printing comprise monomers, predominantly (meth) acrylates, along with photoinitiators and various additives [53]. The filler is used to enhance the resin properties, however, it may affect the degree of conversion (DC) [6]. Franz reported a higher DC in less-filled resins than in highly filled resins [54]. Chen et al. [31] stated that TiO_2_ absorbs UV light, which might negatively affect the curing process, and when the content of TNPs surpasses a critical threshold within the resin matrix, it could have excessively absorbed ultraviolet (UV) light, resulting in voids and the incomplete curing of the resin. This potentially reduces the mechanical properties of the composite resin. 

According to the ASIGA manufacturer, the resin contains inorganic fillers, including TNPs, while the NextDent resin contains some inorganic fillers, is free from TiO_2._ For the ASIGA resin, the addition of the TNPs showed insignificantly higher flexural strength values compared with unmodified TNPs in terms of the respective PCT except at 2% at 60 min, while for the NextDent resin, this increase was significant. Based on the SEM findings, the change in the surface topography with TNP addition from a smooth to irregular background, especially with the NextDent resin, confirms the change in the mechanical performance of the nanocomposite 3D-printable resin. This increase may be related to the proper bonding of the silanated TNPs and the good distribution (no cluster formations) of TNPs within the resin matrix, in addition to the addition of TNPs at low concentrations. Azmy et al. [55] added 3% and 7% TNPs to PMMA and reported that the addition of more than 3% TNPs decreased the strength and recommended low concentrations [55]. Chen et al. [52] found that the TNPs increased the flexural properties of 3D-printed resin with low content, and when the TNPs increased up to 2%, there was no significant difference in the unmodified group. This might explain the results of the insignificant increase in the FS for the Asiga resin group because it already included titanium dioxide in its ingredients; therefore, after the addition of the TNPs, the total percent might have exceeded 2%.

Other studies [40,41] found opposite results of a decreased FS with the addition of TNPs. Pai et al. [56] added TNPs at different concentrations of 0.5%, 1%, 2%, and 3% and found that the flexural strength of PMMA decreased with the addition of TNPs with a gradual decline with increasing concentrations of nanoparticles. In contrast, the study by Bdelraouf et al. [57] showed an increase in the FS with 5% TNPs, which may be due to the resin type used: autopolymerized resin. The explanation for the decreased FS strength with a high percentage of TNPs could be based on the fact that the amount of TNPs affects the DC in addition to the UV light absorption by TiO_2,_ subsequently affecting the mechanical properties. Due to the variation between the materials and printing technologies, NextDent showed a higher FS and hardness when compared with ASIGA. After TNP incorporation, the strength of the 3D-printable resin is material-dependent, and low concentrations are recommended.

Bayarsaikhan et al. [49] reported that the elastic modulus of specimens have the same tendency and behavior as the flexural strength findings; however, this does not align with our findings, in which the elastic modulus results did not show a similar tendency to the flexural strength. For both the unmodified resins, although the results varied, the elastic modulus was not significantly affected by the PCT. This finding contradicts the outcomes of Jindal et al. [58], who studied the mechanical properties of 3D-printable clear aligners under various PCT temperatures.

Based on the present findings, the elastic modulus values for all the groups exceeded 2000 MPa (the recommended ISO ISO 20795-1:2013 [46]). For both resins, TNPs did not show a significant impact on the elastic modulus; although, 1% TNP showed a better performance than 2% TNP, except at 90 min with 1% TNPs, which showed an increased elastic modulus in both the resins. Regarding the PCT, the 2% TNP concentration showed decreased values in comparison to the 1% TNP concentration. This result revealed that increased TNPs decreased the elastic modulus. This may be attributed to the presence of TNPs, which necessitated a lesser displacement for sample fracture [50]. In terms of the material type, the elastic modulus increased for the NextDent resin with added TNPs at a 90 min PCT. This variation might result from the different material ingredients. However, more investigations are needed that test the flexural modulus.

The hardness of the unmodified resins increased with the PCT, which matches that of the flexural strength. It was verified that extended post-curing durations lead to a higher degree of conversion, reduced residual monomer content and subsequently enhanced hardness [50]. The residual monomer acts as a plasticizer, weakening the resin [48]. Hence, a direct correlation between the resin hardness and the post-curing time was stated as demonstrated by Aati et al. [59] and Al-Dulaijan et al. [23]. Additionally, this study agrees with Lee et al. [60], who proved that the high hardness value correlating with the post-curing time might result from the rate of polymerization and the degree of conversion. 

Kim et al. [60] demonstrated that following a 15 min post-curing period, there was a gradual but relatively modest increase in the Vickers hardness with time. The initial improvement in the hardness during this short post-curing period is likely due to enhancements in the physical properties of the outermost layer at the outset of the post-curing process [50]. This characteristic was also reflected in the findings concerning the degree of conversion. It was observed that the degree of conversion exhibits slow improvement during the post-curing period. The increase in the hardness was confirmed and explained by Kim et al. [51]. Once the 3D printing process is completed, it is common for the physical properties, such as the mechanical strength, to be insufficient. Therefore, it becomes critically important to enhance these properties by addressing the anisotropy and increasing the polymerization rate through post-curing after 3D printing, as highlighted in a previous study [61]. When the degree of polymerization is primarily enhanced in the superficial layers while the overall strength remains unaffected, it becomes evident that a sufficient post-curing time is required to address this situation effectively [62]. Bayarsaikhan et al. [49] demonstrated an increase in the hardness as the PCT increased, in agreement with the present results.

For the ASIGA resin, the hardness was not affected by the addition of a 1% TNP concentration, but a 2% TNP concentration decreased the hardness, while the NextDent resin showed an insignificant change with TNPs in terms of their respective PCT. This confirms the effect of the PCT on the specimen surface and masks the effect of the TNPs. The decrease in hardness may be due to the effect of the NPs on the degree of conversion [46]. According to Gad et al. [21], the incorporation of nanoparticles (NPs) into the 3D-printed resins led to a negligible reduction in the hardness values when compared to the unmodified resin. Al-Dwairi et al. [63] observed a lower hardness of 3D-printed resins when compared to heat-polymerized PMMA. The surface hardness appeared to be influenced negatively by the presence of residual monomers, as their concentration could affect the hardness. Momper et al. [64] studied the influence of NPs on the 3D printing and post-curing of nanocomposite resins and found that the incorporation of NPs can affect the degree of the conversion and the curing behavior of the resin.

The current study found no significant difference in the mean hardness values of the 3D-printable denture base resins modified with TNPs compared to those of the control, except for the Asiga-2% TNP resins, for which the hardness was significantly decreased. The effect of TNPs on the hardness of 3D-printed resin has not been tested extensively before [29], which makes comparison to the present results difficult. Nevertheless, previous studies have evaluated the effect of TNPs on the hardness of PMMA denture base materials and have reported higher hardness values than the unmodified group [65]. However, the increased TNP amount beyond the saturation level adversely affected the degree of conversions and increased the amount residual monomers, which act as plasticizers [59]. This is also consistent with the findings of Chen et al. [31] and Altarazi et al. [30], who reported a high hardness in low concentrations when compared to high concentrations and attributed the main cause to the degree of conversion. Likewise, Alhotan et al. [66] concluded that, with an increase in the nanofiller content, the surface hardness of the reinforced PMMA also increased. They attributed this observation to either the uniform dispersion of the TNPs within the matrix or the utilization of a silane coupling agent, which enhanced the chemical bonding between the PMMA matrix and the fillers [66]. The TNP concentration was lower than that tested in previous studies, as well as the denture base material (PMMA), which might be the reason for the variation in the hardness values with the addition of the TNPs.

Among the aspects that could affect the properties of 3D-printed resin is the degree of conversion, which is used as a guide for the polymerization rate. A higher DC results in a decreased residual monomer content and the improved mechanical characteristics of the printed object. During the printing process, the printed objects are not completely polymerized inside the printer, and post-curing is required to promote cross-linking, improve the biocompatibility, increase the DC, and consume any residual photoinitiator [67]. Reymus et al. [68] reported that with an increased post-curing time, the degree of conversion increased. Kim et al. [51] found that the strength of 3D-printed resins increased when the PCT was more than 60 or 90 min. This proves that the concept of the positive effect of the PCT on the superficial layer only had a small effect on the resin inside [59,60]. Accordingly, an adequate PCT should be applied to the printed objects to ensure consistent polymerization through all the layers (outer and inner) of the 3D-printed objects [69]. This notion is supported by the results of the flexural modulus analysis, which indicated that the group subjected to a shorter post-curing time exhibited very low values, while the values increased as the post-curing duration increased.

Three-dimensionally printed resin properties can be affected by different factors [6], such as the incorporation of nanoparticles and post-curing conditions. The strength of the nanocomposite is affected by the nanoparticle concentration; therefore, various concentrations must be considered in future investigations. Additionally, an increased PCT is recommended due to its positive impact on unmodified and nanocomposite 3D-printed resins. The variations in the results of some tested properties, such as the elastic modulus, require caution in explaining the results. More investigations are needed with different resin brands, different NPs and concentrations, and with different tested properties, especially DC tests.

The strengths of this study were the testing of two different types of 3D-printable resins and subjecting the specimens to artificial aging by thermal cycling. However, testing one type of NP is considered a limitation. Another limitation is the lack of exposure to other oral conditions, such as saliva, pH variation, masticatory force, denture disinfection procedures, and beverage intake. Therefore, further in vivo or in vitro investigations are needed in conditions simulating oral conditions. In addition, testing specimens of real denture configuration is required to confirm the present results.

## 5. Conclusions

The post-curing time has a positive effect on the flexural strength and hardness of 3D-printed resin, and an increased PCT is recommended. The flexural strength was increased by the addition of TNPs to 3D-printable denture base resin, while the elastic modulus and hardness of the printed nanocomposite decreased with TNP addition (2%). The mechanical properties of the 3D-printed nanocomposite were affected by the combined effect of multiple factors including the material type, TNP concentration, and PCT. However, further investigations are needed with other parameters considering the mechanical, biological, and physical characteristics of the introduced nanocomposites before clinical recommendations.

## Figures and Tables

**Table 1:** Summary of 3D-printed materials specifications and details of printing parameters and printing process.

	(ASIGA)	NextDent
Materials/Brand name	DentaBASE ASIGA, Erfurt, Germany	Denture 3D+ NextDent B.V., Soesterberg, The Netherlands
Composition	Ethoxylated bisphenol A dimethacrylate 7,7,9 (or 7,9,9)-trimethyl-4,13-dioxo-3,14-dioxa-5,12-diazahexadecane-1,16-diyl bismethacrylate 2- hydroxyethyl methacrylate silicon dioxide diphenyl (2,4,6-trimethylbenzoyl) phosphine oxide titanium dioxide	Ester-based monomer; Bisacylphosphine oxide (BAPO) phenylbis (2,4,6-trimethylbenzoyl)-phosphine oxide (Omnirad 819)
Printer	ASIGA MAX™	NextDent 5100
Printing technology	DLP	SLA
Printing layer thickness	50 µm	50 µm
Printing orientation	90 degrees	90 degrees
Post-curing machine	ASIGA-Flash	LC-D Print Box
Post-curing time	15 min, 60 min, and 90 min	15 min, 60 min, and 90 min
Post-curing temperature	60 °C	60 °C

**Table 2:** Mean, SD, and pairwise significance between 3D-printed resins for all tested properties.

Properties	Materials	TNPs%	Time			*p*-Value
			15 min	60 min	90 min	
Flexural strength	ASIGA	0%	73.3 (2.6)	81.1 (2.4) ^a^	87.3 (3.2)	0.000 *
		1%	76.0 (3.3) ^A^	81.5 (2.8) ^a,A^	91.4 (9.8)	0.000 *
		2%	77.7 (9.5)	86.6 (2.9) ^A^	90.4 (2.9) ^A^	0.000 *
		*p*-value	0.281	0.000 *	0.323	
	NextDent	0%	72.3 (2.8)	79.9 (2.6)	85.2 (1.8)	0.000
		1%	78.1 (3.2)	87.1 (1.6) ^a^	92.0 (2.4) ^a^	0.000
		2%	83.2 (4.3) ^A^	87.3 (3.4) ^a,A^	93.6 (4.1) ^a^	0.000
		*p*-value	0.000 *	0.000 *	0.000 *	
Elastic modulus	ASIGA	0%	3511.0 (132.5)	3641.3 (220.9)	3585.7 (156.6) ^a,b^	0.262
		1%	3534.6 (236.7) ^A,B^	3356.4 (260.8) ^A^	3637.9 (187.3) ^a,B^	0.035 *
		2%	3406.8 (285.6)	3583.2 (391.5)	3356.4 (304.8) ^b^	0.290
		*p*-value	0.421	0.10	0.022 *	
	NextDent	0%	3579.9 (399.6) ^a^	3726.1 (436.2) ^a^	3928.8 (509.7)	0.239
		1%	3548.6 (229.1) ^a^	4088.3 (187.6) ^b,A^	4243.6 (173.2) ^A^	0.000 *
		2%	3181.0 (259.9)	3779.9 (166.6) ^a,b^	4062.5 (92.9)	0.000 *
		*p*-value	0.012 *	0.020 *	0.10	
Hardness	ASIGA	0%	15.2 (5.4) ^A^	16.8 (2.8) ^A,B^	19.7 (2.1) ^a,B^	0.038 *
		1%	15.9 (2.2)	15.3 (6.2)	16.4 (6.4) ^a,b^	0.892
		2%	11.9 (3.4)	12.4 (3.9)	14.9 (2.5) ^b^	0.122
		*p*-value	0.067	0.105	0.043 *	
	NextDent	0%	15.5 (4.9) ^A^	16.9 (3.9) ^A,B^	20.7 (3.7) ^B^	0.029 *
		1%	16.5 (5.7)	18.8 (5.2)	18.9 (4.4)	0.516
		2%	16.6 (9.0)	18.6 (6.4)	18.5 (2.1)	0.741
		*p*-value	0.918	0.683	0.353	

* Statistically significant at 0.05 level of significance. Same small superscript letter per material indicates insignificant pairwise comparison in terms of concentration effect for each time. Same capital letter per raw figure indicates insignificant pairwise comparison in terms of time effect for each concentration.

**Table 3:** Three-way ANOVA results for all tested properties and in terms of resin type, NPs %, and post-curing time.

Tested Properties	Source	Type III Sum of Squares	df	Mean Square	F	Sig.
Flexural strength	Corrected Model	7087.190 ^a^	17	416.894	22.815	<0.001 *
	Intercept	1,257,044.898	1	1,257,044.898	68,792.949	<0.001 *
	time * concen	55.998 21.409 197.040 105.164	4 2 2 4	14.000	0.766	0.549
	time * resin concen * resin			10.705 98.520 26.291	0.586 5.392	0.558
						0.005 *
	time * concen * resin				1.439	0.224
	Error	2960.206	162	18.273		
	Total	1,267,092.293	180			
	Corrected Total	10,047.395	179			
a. R Squared = 0.705 (Adjusted R Squared = 0.674)						
Elastic modulus	Corrected Model	13,229,873.357 ^a^	17	778,227.845	9.940	<0.001 *
	Intercept	2,401,859,264.539	1	2,401,859,264.5	30,676.787	<0.001 *
	time * concen	539,974.604	4	134,993.651	1.724	0.147
	time * resin concen * resin	2,767,384.066	2	1,383,692.03	17.673 4.322	<0.001 * 0.015 *
		676,772.858	2	338,386.429		
	time * concen * resin	1,116,207.931	4	279,051.983	3.564	0.008 *
	Error	12,683,896.631	162	78,295.658		
	Total	2,427,773,034.527	180			
	Corrected Total	25,913,769.988	179			
a. R Squared = 0.511 (Adjusted R Squared = 0.459)						
Hardness	Corrected Model	921.092 ^a^	17	54.182	2.325	0.003 *
	Intercept	49,926.694	1	49,926.694	2142.394	<0.001 *
	time * concen	69.218	4	17.304	0.743	0.564
	time * resin	16.508	2	8.254	0.354	0.702
	concen * resin	145.005	2	72.502	3.111	0.047 *
	time * concen * resin	26.349	4	6.587	0.283	0.889
	Error	3775.274	162	23.304		
	Total	54,623.060	180			
	Corrected Total	4696.366	179	Corrected Total		
a. R Squared = 0.196 (Adjusted R Squared = 0.112)						

* Statistically significant at 0.05 level of significance.

**Table 4:** Resin type pairwise comparison in terms of concentrations at fixed post-curing time.

Tested Properties	Concentration	Resin	Time		
			15 min	60 min	90 min
Flexural strength	0%	ASIGA	73.34 (2.6)	81.13 (2.4)	87.3 (3.2)
		NextDent	72.34 (2.8)	79.88 (2.6)	85.2 (1.9)
	P		0.421	0.284	0.082
	1%	ASIGA	76.03 (3.29)	81.49 (2.8)	91.36 (9.7)
		NextDent	78.08(3.2)	87.1 (1.6)	92.03 (2.4)
	P		0.721	0.000 *	0.837
	2%	ASIGA	77.7 (9.5)	86.6 (2.9)	90.4 (2.9)
		NextDent	83.3 (4.3)	87.3 (3.4)	93.6 (4.1)
	P		0.110	0.610	0.064
Elastic Modulus	0%	ASIGA	3511.0 (132.5)	3641.29 (220.9)	3585.7 (156.6)
		NextDent	3579.9 (399.6)	3726.13 (436.2)	3928.8 (509.8)
	P		0.611	0.59	0.057
	1%	ASIGA	3534.56 (236.7)	3356.47 (260.8)	3637.8 (187.3)
		NextDent	3548.58 (229.1)	4088.35 (187.6)	4243.6 (173.3)
	P		0.894	<0.001 *	<0.001 *
	2%	ASIGA	3406.8 (285.6)	3583.2 (391.5)	3356.3 (304.8)
		NextDent	3181.0 (259.9)	3779.9 (166.5)	4062.5 (92.9)
	P		0.081	0.161	0.000 *
Hardness	0%	ASIGA	15.23 (5.4)	16.48 (2.8)	19.68 (2.1)
		NextDent	15.49 (4.9)	16.95 (3.9)	20.69 (3.7)
	P		0.912	0.943	0.464
	1%	ASIGA	15.9 (2.2)	15.3 (6.2)	16.4 (6.3)
		NextDent	16.6 (5.7)	18.8 (5.2)	18.9 (4.5)
	P		0.757	0.186	0.320
	2%	ASIGA	11.9 (3.4)	12.4 (3.9)	14.9 (2.5)
		NextDent	16.6 (9.0)	18.6 (6.4)	18.4 (2.1)
	P		0.143	0.017 *	0.003 *

* Statistically significant at 0.05 level of significance.

## Data Availability

The data are available upon request via email or phone to the corresponding author.
